# Inequalities for higher order differences of the logarithm of the overpartition function and a problem of Wang–Xie–Zhang

**DOI:** 10.1007/s40993-022-00420-y

**Published:** 2022-12-19

**Authors:** Gargi Mukherjee

**Affiliations:** Institute for Algebra, Johannes Kepler University, Altenberger Strasse 69, 4040 Linz, Austria

**Keywords:** Overpartition, $\log$-concavity, Finite difference, Primary 05A20, 11B68

## Abstract

Let ${\overline{p}}(n)$ denote the overpartition function. In this paper, our primary goal is to study the asymptotic behavior of the finite differences of the logarithm of the overpartition function, i.e., $(-1)^{r-1}\Delta ^r \log {\overline{p}}(n)$, by studying the inequality of the following form

$$\begin{aligned} \log \Bigl (1+\dfrac{C(r)}{n^{r-1/2}}-\dfrac{1+C_1(r)}{n^{r}}\Bigr ){} & {} <(-1)^{r-1}\Delta ^r \log {\overline{p}}(n) \\ {}{} & {} <\log \Bigl (1+\dfrac{C(r)}{n^{r-1/2}}\Bigr )\ \text {for}\ n \ge N(r), \end{aligned}$$

where $C(r), C_1(r), \text {and}\ N(r)$ are computable constants depending on the positive integer *r*, determined explicitly. This solves a problem posed by Wang, Xie and Zhang in the context of searching for a better lower bound of $(-1)^{r-1}\Delta ^r \log {\overline{p}}(n)$ than 0. By settling the problem, we are able to show that

$$\begin{aligned} \lim _{n\rightarrow \infty }(-1)^{r-1}\Delta ^r \log {\overline{p}}(n) =\dfrac{\pi }{2}\Bigl (\dfrac{1}{2}\Bigr )_{r-1}n^{\frac{1}{2}-r}. \end{aligned}$$

## Introduction

An overpartition of a positive integer *n* is a nonincreasing sequence of positive integers whose sum is *n* in which the first occurrence of a number may be overlined, ${\overline{p}}(n)$ denotes the number of overpartitions of *n*, and we define ${\overline{p}}(0)=1$. For example, there are 8 overpartitions of 3 enumerated by $3, {\overline{3}}, 2+1, {\overline{2}}+1, 2+{\overline{1}}, {\overline{2}}+{\overline{1}},1+1+1, {\overline{1}}+1+1$. A thorough study of the overpartition function started with the work of Corteel and Lovejoy [1], although it has been studied under different nomenclature that dates back to MacMahon. Similar to the Hardy-Ramanujan-Rademacher formula for the partition function (cf. [2, 3]), Zuckerman’s [4] formula for ${\overline{p}}(n)$ states that

1.1 $$\begin{aligned} {\overline{p}}(n)=\frac{1}{2\pi }\underset{2 \not \mid k}{\sum _{k=1}^{\infty }}\sqrt{k}\underset{(h,k)=1}{\sum _{h=0}^{k-1}}\dfrac{\omega (h,k)^2}{\omega (2h,k)}e^{-\frac{2\pi i n h}{k}}\dfrac{d}{dn}\biggl (\dfrac{\sinh \frac{\pi \sqrt{n}}{k}}{\sqrt{n}}\biggr ), \end{aligned}$$

where

$$\begin{aligned} \omega (h,k)=\text {exp}\Biggl (\pi i \sum _{r=1}^{k-1}\dfrac{r}{k}\biggl (\dfrac{hr}{k}-\biggl \lfloor \dfrac{hr}{k}\biggr \rfloor -\dfrac{1}{2}\biggr )\Biggr ) \end{aligned}$$

for $(h,k)\in \mathbb {Z}_{\ge 0}\times \mathbb {Z}_{\ge 1}$. Engel [5] determined an error term for ${\overline{p}}(n)$ and found that

1.2 $$\begin{aligned} {\overline{p}}(n)=\frac{1}{2\pi }\underset{2 \not \mid k}{\sum _{k=1}^{N}}\sqrt{k}\underset{(h,k)=1}{\sum _{h=0}^{k-1}}\dfrac{\omega (h,k)^2}{\omega (2h,k)}e^{-\frac{2\pi i n h}{k}}\dfrac{d}{dn}\biggl (\dfrac{\sinh \frac{\pi \sqrt{n}}{k}}{\sqrt{n}}\biggr )+R_{2}(n,N), \end{aligned}$$

where

1.3 $$\begin{aligned} \bigl |R_{2}(n,N)\bigr |< \dfrac{N^{5/2}}{\pi n^{3/2}} \sinh \biggl (\dfrac{\pi \sqrt{n}}{N}\biggr ), \end{aligned}$$

similar to the work done by Lehmer [6] in order to obtain an error bound for the partition function *p*(*n*).

A positive sequence $\{a_n\}_{n \ge 0}$ is said to be $\log$-concave (resp. $\log$-convex) if for all $n \ge 1$, $a^2_n\ge a_{n-1}a_{n+1}$ (resp. $a^2_n\le a_{n-1}a_{n+1}$), and it is said to be strictly $\log$-concave (resp. strictly $\log$-convex) if the inequality is strict.

Using the notations above, Engel’s result [5] actually states that $\{{\overline{p}}(n)\}_{n \ge 1}$ is $\log$-concave. In fact, if one defines ${\overline{p}}(0):=1$, then $\{{\overline{p}}(n)\}_{n \ge 0}$ is actually also $\log$-concave. Engel proved that $\{{\overline{p}}(n)\}_{n \ge 4}$ is strictly $\log$-concave by using the asymptotic formula (1.2) with $N=3$, and the error bound (1.3). Prior to Engel’s work on overpartitions, the $\log$-concavity of the partition function *p*(*n*) and its associated inequalities has been studied in a wider spectrum, details can be found in [7–9]. On the other hand, Liu and Zhang [10] proved a family of inequalities for the overpartition function. Higher order $\log$-concavity and $\log$-convexity for the overpartition function has been studied in [11, 12] respectively.

Chen, Guo and Wang [13] introduced the notion of ratio $\log$-convexity of a sequence and established that ratio $\log$-convexity implies $\log$-convexity under a certain initial condition. A sequence $\{a_n\}_{n \ge k}$ is called ratio $\log$-convex if $\{a_{n+1}/a_n\}_{n \ge k}$ is $\log$-convex or, equivalently, for $n \ge k+1$,

$$\begin{aligned} \Delta ^3 \log a_{n-1}=\log a_{n+2}-3\log a_{n+1}+3\log a_n-\log a_{n-1}\ge 0, \end{aligned}$$

where $\Delta$ be the difference operator defined by $\Delta f(n)=f(n+1)-f(n)$. Chen, Guo, and Wang relates the ratio $\log$-convexity of a sequence, say $\{a_n\}_{n \ge k}$, with strict $\log$-convexity of the associated sequence $\{\root n \of {a_n}\}_{n \ge k}$ stated in the following theorem.

### Theorem 1.1

[13, Theorem 3.6] Let *k* be a positive integer. If a sequence $\{a_n\}_{n \ge k}$ is ratio $\log$-convex and

$$\begin{aligned} \dfrac{\root k+1 \of {a_{k+1}}}{\root k \of {a_{k}}}<\dfrac{\root k+2 \of {a_{k+2}}}{\root k+1 \of {a_{k+1}}}, \end{aligned}$$

then the sequence $\{\root n \of {a_n}\}_{n \ge k}$ is strictly $\log$-convex.

Similar to the work done by Chen et al. [8] for *p*(*n*), Wang, Xie and Zhang [14] proved the following two theorems.

### Theorem 1.2

[14, Theorem 3.1] For each $r\ge 1$, there exists a positive number *n*(*r*) such that for all $n \ge n(r)$,

$$\begin{aligned} (-1)^{r-1}\Delta ^r\log {\overline{p}}(n)>0. \end{aligned}$$

### Theorem 1.3

[14, Theorem 4.1] For each $r\ge 1$, there exists a positive number *n*(*r*) such that for all $n \ge n(r)$,

$$\begin{aligned} (-1)^{r-1}\Delta ^r\log {\overline{p}}(n)<\dfrac{\pi }{2}\Bigl (\dfrac{1}{2}\Bigr )_{r-1}\dfrac{1}{n^{r-\frac{1}{2}}}, \end{aligned}$$

where $$\left( \alpha \right) _{r}:=\alpha \cdot (\alpha +1)\cdots (\alpha +r-1)$$.

### Remark 1.4

Following Theorem 1.3, we observe that $\log$-concavity and ratio $\log$-convexity for ${\overline{p}}(n)$ correspond to the cases $r=2$ and $r=3$ respectively.

Wang, Xie, and Zhang raised the following question:

### Problem 1.5

[14, Problem 3.4] Does there exist a positive number A such that

$$\begin{aligned} n^{r-1/2}(-1)^{r-1}\Delta ^r \log {\overline{p}}(n)>A, \end{aligned}$$

for any $r\ge 1$ and all sufficiently large *n*?

In other words, their problem reads “*Moreover, we seek a sharp lower bound for *$(-1)^{r-1}\Delta ^r \log {\overline{p}}(n)$”.

The main motivation of this paper is to give an affirmative answer to the Problem 1.5 in Theorems 1.6 and 1.8. This in turn clarifies the asymptotic growth of $(-1)^{r-1}\Delta ^r \log {\overline{p}}(n)$, see Corollary 1.9. In Corollaries 1.10 and 1.11, we recover the $\log$-concavity and its (shifted) companion inequality respectively.

### Theorem 1.6

For $$n \ge 26$$,

1.4 $$\begin{aligned} \log \Bigl (1+\dfrac{\pi }{2\sqrt{n}}\Bigr )<\Delta \log {\overline{p}}(n)<\log \Bigl (1+\dfrac{\pi }{2\sqrt{n}}+\dfrac{\pi ^2}{40n}\Bigr ). \end{aligned}$$

### Definition 1.7

For $r \ge 2$, we define

1.5 $$\begin{aligned} N_0(m):= & {} {\left\{ \begin{array}{ll} 1, &{}\quad \text {if}\ m=1,\\ 2m \log m-m \log \log m, &{} \quad \text {if}\ m \ge 2, \end{array}\right. } \end{aligned}$$

1.6 $$\begin{aligned} N_1(r):= & {} \max \Biggl \{85, \Biggl \lceil \dfrac{4}{\pi ^2}N^2_0(2r+2)\Biggr \rceil \Biggr \},\end{aligned}$$

1.7 $$\begin{aligned} C(r):= & {} \dfrac{\pi }{2}\Bigl (\dfrac{1}{2}\Bigr )_{r-1},\end{aligned}$$

1.8 $$\begin{aligned} C_1(r):= & {} (r-1)!+4r^2 C(r),\end{aligned}$$

1.9 $$\begin{aligned} C_2(r):= & {} \sum _{k=0}^{2r-2}\dfrac{1}{(k+1)\pi ^{k+1}}\Bigl (\dfrac{k+1}{2}\Bigr )_r \dfrac{1}{r^k}+\dfrac{r}{10^r},\end{aligned}$$

1.10 $$\begin{aligned} N_2(r):= & {} \Biggl \lceil \Biggl (\dfrac{1+C_1(r)}{C(r)}\Biggr )^2\Biggr \rceil ,\end{aligned}$$

1.11 $$\begin{aligned} N_3(r):= & {} \max \Biggl \{N_1(r),2r^2, \Biggl \lceil \Biggl (\dfrac{2^{r+1}\Bigl (C_2(r)+1\Bigr )}{(r-1)!}\Biggr )^2\Biggr \rceil , \Biggl \lceil \root r-1 \of {\Biggl (\dfrac{2^r C(r)^2}{(r-1)!}\Biggr )} \Biggr \rceil \Biggr \},\end{aligned}$$

1.12 $$\begin{aligned} and N(r):= & {} \max \Bigl \{N_2(r), N_3(r)\Bigr \}. \end{aligned}$$

### Theorem 1.8

For $$r \in \mathbb {Z}_{\ge 2}$$ and $$n \ge N(r)$$,

1.13 $$\begin{aligned} 0<\log \Bigl (1+\dfrac{C(r)}{n^{r-1/2}}-\dfrac{1+C_1(r)}{n^{r}}\Bigr )<(-1)^{r-1}\Delta ^r \log {\overline{p}}(n) <\log \Bigl (1+\dfrac{C(r)}{n^{r-1/2}}\Bigr ), \nonumber \\ \end{aligned}$$

where *C*(*r*) and $C_1(r)$ are given in (1.7)–(1.8).

### Corollary 1.9

For $$r \in \mathbb {Z}_{\ge 1}$$,

1.14 $$\begin{aligned} \lim _{n\rightarrow \infty } n^{r-1/2}(-1)^{r-1}\Delta ^r \log {\overline{p}}(n)= \dfrac{\pi }{2}\Bigl (\dfrac{1}{2}\Bigr )_{r-1}. \end{aligned}$$

### Proof

Multiplying both sides of (1.4) (resp. (1.13)) by $\sqrt{n}$ (resp. by $n^{r-1/2}$) and taking limits as *n* tends to infinity, we obtain (1.14). $\square$

### Corollary 1.10

[5, Theorem 1.2] For $$n \ge 4$$, $${\overline{p}}(n)^2\ge {\overline{p}}(n-1){\overline{p}}(n+1)$$.

### Proof

Observe that $N(2)=344$ and from the lower bound of (1.13), we observe that $\{{\overline{p}}(n)\}_{n \ge 344}$ is $\log$-concave and for the remaining cases $5 \le n \le 343$, we confirm by numerical checking in Mathematica. $\square$

Consider the shifted version of Corollary 1.10, namely, for $n\ge 3$, ${\overline{p}}(n+1)^2\ge {\overline{p}}(n){\overline{p}}(n+2)$. Analogous to [10, Equation (1.6)], we obtain the (shifted) companion inequality in the following form.

### Corollary 1.11

For $$n \ge 1$$,

1.15 $$\begin{aligned} \dfrac{{\overline{p}}(n)}{{\overline{p}}(n+1)}\Bigl (1+\dfrac{\pi }{4n^{3/2}}\Bigr )>\dfrac{{\overline{p}}(n+1)}{{\overline{p}}(n+2)}. \end{aligned}$$

### Proof

Using (1.13) with $r=2$ directly gives (1.15). $\square$

### Corollary 1.12

For $$n \ge 18$$, $$\Delta ^3\log {\overline{p}}(n-1)>0$$.

### Proof

Applying (1.13) with $r=3$, we observe that for $n \ge 1486=N(3)$, $\Delta ^3\log {\overline{p}}(n)>0$, which is equivalent to say that for $n \ge 1487$, $\Delta ^3\log {\overline{p}}(n-1)>0$. For the remaining cases $18 \le n \le 1486$, we confirm the inequality $\Delta ^3\log {\overline{p}}(n-1)>0$ by numerical checking in Mathematica. $\square$

Define $$r(n):=\root n \of {{\overline{p}}(n)}$$.

### Corollary 1.13

[11, Corollary 1.3] For $$n \ge 4$$, $$r(n)^2< r(n-1)r(n+1)$$.

### Proof

From Corollary 1.12, we have $\{{\overline{p}}(n)\}_{n \ge 18}$ is ratio $\log$-convex. Now applying Theorem 1.1 with $k=18$, and checking numerically

$$\begin{aligned} \dfrac{\root 19 \of {{\overline{p}}(19)}}{\root 18 \of {{\overline{p}}(18)}}<\dfrac{\root 20 \of {{\overline{p}}(20)}}{\root 19 \of {{\overline{p}}(19)}}, \end{aligned}$$

we conclude that $\{r(n)\}_{n \ge 18}$ is strictly $\log$-convex; i.e., for all $n \ge 18$, $r(n)^2< r(n-1)r(n+1)$, and for the remaining cases $4\le n \le 17$, we confirm by numerical checking in Mathematica. $\square$

This paper is organized as follows. A preliminary setup for decomposing $(-1)^{r-1}\Delta ^r \log {\overline{p}}(n)$ $=H_r+G_r$ (cf. see (2.4), (2.5), and (2.6)), as done in [14], and estimations for both $H_r$ and $G_r$ are given in Sect. 2. Proofs of Theorems 1.6 and 1.8 are given in Sect. 3.

## preliminary lemmas

Following the notations given in Engel [5] and Wang et al. [14], split ${\overline{p}}(n)$ as

2.1 $$\begin{aligned} {\overline{p}}(n)={\widehat{T}}(n) \Biggl (1+\dfrac{{\widehat{R}}(n)}{{\widehat{T}}(n)}\Biggr ), \end{aligned}$$

where

2.2 $$\begin{aligned} {\widehat{T}}(n)= & {} \dfrac{1}{8n}\Bigl (1-\dfrac{1}{\widehat{\mu }(n)}\Bigr )e^{\widehat{\mu }(n)} \end{aligned}$$

2.3 $$\begin{aligned} \text {and} {\widehat{R}}(n)= & {} \dfrac{1}{8n}\Bigl (1+\dfrac{1}{\widehat{\mu }(n)}\Bigr )e^{-\widehat{\mu }(n)}+R_2(n,3) \end{aligned}$$

with $$\widehat{\mu }(n)=\pi \sqrt{n}$$.

### Remark 2.1

The splitting for ${\overline{p}}(n)$ used here is actually slightly different from what is found in [5, 14].

Taking the logarithm on both sides of (2.1) and plugging the definitions from (2.2)–(2.3), we obtain

$$\begin{aligned} \log {\overline{p}}(n)=\log \dfrac{\pi ^2}{8}-3 \log \widehat{\mu }(n)+\log (\widehat{\mu }(n)-1)+\widehat{\mu }(n)+\log \ \Biggl (1+\dfrac{{\widehat{R}}(n)}{{\widehat{T}}(n)}\Biggr ). \end{aligned}$$

Therefore,

2.4 $$\begin{aligned} (-1)^{r-1}\Delta ^r \log {\overline{p}}(n)=H_r+G_r, \end{aligned}$$

where

2.5 $$\begin{aligned} H_r= & {} (-1)^{r-1}\Delta ^r (-3 \log \widehat{\mu }(n)+\log (\widehat{\mu }(n)-1)+\widehat{\mu }(n)) \end{aligned}$$

2.6 $$\begin{aligned} G_r= & {} (-1)^{r-1}\Delta ^r \log \ \Biggl (1+\dfrac{{\widehat{R}}(n)}{{\widehat{T}}(n)}\Biggr ). \end{aligned}$$

Then we have that for $r \ge 1$,

2.7 $$\begin{aligned} H_r-|G_r| \le (-1)^{r-1}\Delta ^r \log {\overline{p}}(n) \le H_r+|G_r|. \end{aligned}$$

To estimate the bounds for $(-1)^{r-1}\Delta ^r \log {\overline{p}}(n)$, we need to establish bounds for $H_r$ and $|G_r|$. Our first goal is to determine a bound for $|G_r|$ for $r \ge 1$ and then we further proceed with $H_r$ but splitting into cases, namely, for $r=1$ and $r \ge 2$.

### Lemma 2.2

[12, Lemma 2.1] For any integer $m\ge 1$ and $x \ge N_0(m)$,

$$\begin{aligned} x^me^{-x}<1, \end{aligned}$$

where $N_0(m)$ is defined in (1.5).

Recall that $N_1(r)= \max \Biggl \{85, \Biggl \lceil \dfrac{4}{\pi ^2}N^2_0(2r+2)\Biggr \rceil \Biggr \}$ (cf. (1.6)).

### Lemma 2.3

For all $n \ge N_1(r)$ and $r \ge 1$,

2.8 $$\begin{aligned} |G_r| < \dfrac{1}{n^{r+1}}. \end{aligned}$$

### Proof

Define ${\widehat{e}}(n):= \frac{{\widehat{R}}(n)}{{\widehat{T}}(n)}$. From the definition of ${\widehat{R}}(n)$ and ${\widehat{T}}(n)$ (cf. Equations (2.2)–(2.3)), we have

2.9 $$\begin{aligned} |{\widehat{e}}(n)|= & {} \dfrac{|{\widehat{R}}(n)|}{|{\widehat{T}}(n)|}\nonumber \\= & {} \Biggl |\dfrac{\frac{1}{8n}\Bigl (1+\frac{1}{\widehat{\mu }(n)}\Bigr ) e^{-\widehat{\mu }(n)}+R_2(n,3)}{\frac{1}{8n}\Bigl (1-\frac{1}{\widehat{\mu }(n)}\Bigr ) e^{\widehat{\mu }(n)}}\Biggr |\nonumber \\< & {} \dfrac{\widehat{\mu }(n)+1}{\widehat{\mu }(n)-1}e^{-2\widehat{\mu }(n)}+ \dfrac{36\sqrt{3}}{\widehat{\mu }(n)-1}e^{-2\widehat{\mu }(n)/3}\nonumber \\{} & {} \quad \ \ \Bigl (\text {using}\ N=3\ \text {in}\ (\ \text {and}\ \sinh (x)<\frac{e^x}{2}\ \text {for}\ x>0\Bigr )\nonumber \\= & {} \dfrac{1}{\widehat{\mu }(n)-1}e^{-\widehat{\mu }(n)/2} \Bigl ((\widehat{\mu }(n)+1)e^{-3\widehat{\mu }(n)/2}+36\sqrt{3}\ e^{-\widehat{\mu }(n)/6}\Bigr ). \end{aligned}$$

Since for all $n \ge 85$,

$$\begin{aligned} (\widehat{\mu }(n)+1)e^{-3\widehat{\mu }(n)/2}+36\sqrt{3}\ e^{-\widehat{\mu }(n)/6}<\frac{1}{2}\ \text {and}\ \frac{1}{\widehat{\mu }(n)-1}<1, \end{aligned}$$

from (2.9), it follows that for all $n \ge 85$,

2.10 $$\begin{aligned} |{\widehat{e}}(n)|<\dfrac{1}{2}\ e^{-\widehat{\mu }(n)/2}. \end{aligned}$$

Therefore, for all $n \ge 85$,

2.11 $$\begin{aligned} |G_r|= & {} \Bigl |(-1)^{r-1}\Delta ^r \log \ (1+{\widehat{e}}(n))\Bigr | \ \ (\text {by}\ (2.6)\nonumber \\= & {} \Biggl |\sum _{i=0}^{r}(-1)^{r-i}\left( {\begin{array}{c}r\\ i\end{array}}\right) \log \ (1+{\widehat{e}}(n+i))\Biggr |\nonumber \\\le & {} \sum _{i=0}^{r} \left( {\begin{array}{c}r\\ i\end{array}}\right) \Bigl |\log \ (1+{\widehat{e}}(n+i))\Bigr |\nonumber \\\le & {} \sum _{i=0}^{r} \left( {\begin{array}{c}r\\ i\end{array}}\right) \dfrac{|{\widehat{e}}(n+i)|}{1-|{\widehat{e}}(n+i)|}\ \ \Bigl (\text {since}\ |\log (1+x)|\le \dfrac{|x|}{1-|x|}\ \text {for}\ |x|<1\Bigr )\nonumber \\\le & {} 2\sum _{i=0}^{r} \left( {\begin{array}{c}r\\ i\end{array}}\right) |{\widehat{e}}(n+i)|\ \Bigl (\text {as}\ \dfrac{x}{1-x}\le 2x\ \text {for}\ 0<x\le \frac{1}{2}\Bigr )\nonumber \\< & {} \sum _{i=0}^{r} \left( {\begin{array}{c}r\\ i\end{array}}\right) e^{-\widehat{\mu }(n+i)/2} \ (\text {by}\ ()\nonumber \\\le & {} \sum _{i=0}^{r} \left( {\begin{array}{c}r\\ i\end{array}}\right) e^{-\widehat{\mu }(n)/2} \ \Bigl (\text {since}\ \{e^{-\widehat{\mu }(n)/2}\}_{n \ge 1}\ \text {is a decreasing sequence}\Bigr )\nonumber \\= & {} 2^r e^{-\widehat{\mu }(n)/2}. \end{aligned}$$

Now applying Lemma 2.2 with $m=2r+2$ and assigning $x \mapsto \frac{\widehat{\mu }(n)}{2}$, it follows that for $n \ge \Biggl \lceil \dfrac{4}{\pi ^2}N^2_0(2r+2)\Biggr \rceil$,

2.12 $$\begin{aligned} e^{-{\widehat{\mu }}(n)/2}< \Bigl (\dfrac{2}{\pi }\Bigr )^{2r+2}\dfrac{1}{n^{r+1}} \implies 2^{r} e^{-{\widehat{\mu }}(n)/2}< \Bigl (\dfrac{2\sqrt{2}}{\pi }\Bigr )^{2r+2}\dfrac{1}{n^{r+1}}<\dfrac{1}{n^{r+1}}. \end{aligned}$$

$$\square $$

Before we state the bounds for $H_r$, we recall the following result due to Odlyzko [15] on the relation between the higher order differences of a smooth function and its derivatives. The following proposition can be proved using elementary techniques such as the mean value theorem.

### Proposition 2.4

Let *r* be a positive integer. Suppose that *f*(*t*) is a function with continuous derivatives for $t\ge 1$, and $(-1)^{k-1}f^{(k)}(t)>0$ for $k \ge 1$. Then for $r\ge 1$, $x\ge 1$,

$$\begin{aligned} (-1)^{r-1}f^{(r)}(x+r)\le (-1)^{r-1}\Delta ^r f(x) \le (-1)^{r-1}f^{(r)}(x). \end{aligned}$$

### Lemma 2.5

For all $$n \ge 1$$,

2.13 $$\begin{aligned} L^{(1)}(n) \le H_1 \le U^{(1)}(n), \end{aligned}$$

where

2.14 $$\begin{aligned} U^{(1)}(n)= & {} \dfrac{\pi }{2\sqrt{n}}-\dfrac{3}{2(n+1)}+\dfrac{\pi }{2\sqrt{n}(\widehat{\mu }(n)-1)} \end{aligned}$$

2.15 $$\begin{aligned} \text {and}\ L^{(1)}(n)= & {} \dfrac{\pi }{2\sqrt{n+1}}-\dfrac{3}{2n}+\dfrac{\pi }{2\sqrt{n+1}(\widehat{\mu }(n+1)-1)}. \end{aligned}$$

### Proof

Equation (2.13) follows immediately by applying Proposition 2.4 on each of the factors in $H_r$ being presented in (2.5) for $r=1$. $\square$

### Lemma 2.6

For $$r \ge 2$$ and $$n \ge 2r^2$$,

2.16 $$\begin{aligned} \dfrac{C(r)}{n^{r-\frac{1}{2}}}-\dfrac{C_1(r)}{n^{r}}< H_r < \dfrac{C(r)}{n^{r-\frac{1}{2}}}-\dfrac{(r-1)!}{2^r\ n^r}+\dfrac{C_2(r)}{n^{r+\frac{1}{2}}}, \end{aligned}$$

where *C*(*r*), $C_1(r)$, and $C_2(r)$ are given by (1.7)–(1.9).

### Proof

Rewrite (2.5) as

2.17 $$\begin{aligned} H_r=(-1)^{r-1}\Delta ^r (\widehat{\mu }(n)-2\log \widehat{\mu }(n)) -\sum _{k=1}^{\infty } (-1)^{r-1}\Delta ^r \Bigl (\dfrac{1}{k \widehat{\mu }(n)^k}\Bigr ) \end{aligned}$$

and applying Proposition 2.4, we get

2.18 $$\begin{aligned} \begin{aligned}&\dfrac{\pi }{2}\Bigl (\dfrac{1}{2}\Bigr )_{r-1}\dfrac{1}{(n+r)^{r-\frac{1}{2}}}-\dfrac{(r-1)!}{n^r}\\&\quad +\sum _{k=1}^{\infty }\dfrac{1}{k\pi ^k}\Bigl (\dfrac{k}{2}\Bigr )_r \dfrac{1}{(n+r)^{r+\frac{k}{2}}}\le H_r\\&\le \dfrac{\pi }{2}\Bigl (\dfrac{1}{2}\Bigr )_{r-1}\dfrac{1}{n^{r-\frac{1}{2}}}-\dfrac{(r-1)!}{(n+r)^r}+\sum _{k=1}^{\infty }\dfrac{1}{k\pi ^k}\Bigl (\dfrac{k}{2}\Bigr )_r \dfrac{1}{n^{r+\frac{k}{2}}}. \end{aligned} \end{aligned}$$

Since for all positive integers *n*, *r* and *k*,

$$\begin{aligned} \sum _{k=1}^{\infty }\dfrac{1}{k\pi ^k}\Bigl (\dfrac{k}{2}\Bigr )_r \dfrac{1}{(n+r)^{r+\frac{k}{2}}}>0. \end{aligned}$$

Therefore,

2.19 $$\begin{aligned}{} & {} \dfrac{\pi }{2}\Bigl (\dfrac{1}{2}\Bigr )_{r-1}\dfrac{1}{(n+r)^{r-\frac{1}{2}}}-\dfrac{(r-1)!}{n^r}< H_r \nonumber \\{} & {} \quad \le \dfrac{\pi }{2}\Bigl (\dfrac{1}{2}\Bigr )_{r-1}\dfrac{1}{n^{r-\frac{1}{2}}}-\dfrac{(r-1)!}{(n+r)^r}+\sum _{k=1}^{\infty }\dfrac{1}{k\pi ^k}\Bigl (\dfrac{k}{2}\Bigr )_r \dfrac{1}{n^{r+\frac{k}{2}}}. \end{aligned}$$

Now we further investigate the lower bound of $H_r$, given in (2.19).

2.20 $$\begin{aligned} H_r\ge & {} \dfrac{\pi }{2}\Bigl (\dfrac{1}{2}\Bigr )_{r-1}\dfrac{1}{(n+r)^{r-\frac{1}{2}}}-\dfrac{(r-1)!}{n^r}\nonumber \\= & {} \dfrac{\pi }{2}\Bigl (\dfrac{1}{2}\Bigr )_{r-1}\dfrac{1}{n^{r-\frac{1}{2}}}\Bigl (1+\dfrac{r}{n}\Bigr )^{-r+\frac{1}{2}}-\dfrac{(r-1)!}{n^r}\nonumber \\= & {} \dfrac{\pi }{2}\Bigl (\dfrac{1}{2}\Bigr )_{r-1}\dfrac{1}{n^{r-\frac{1}{2}}}+\dfrac{\pi }{2}\Bigl (\dfrac{1}{2}\Bigr )_{r-1}\dfrac{1}{n^{r-\frac{1}{2}}} \sum _{m=1}^{\infty }\left( {\begin{array}{c}-\frac{2r-1}{2}\\ m\end{array}}\right) \Bigl (\dfrac{r}{n}\Bigr )^m-\dfrac{(r-1)!}{n^r}. \end{aligned}$$

To bound the infinite series in (2.20), we proceed as follows

2.21 $$\begin{aligned}{} & {} \Biggl |\sum _{m=1}^{\infty }\left( {\begin{array}{c}-\frac{2r-1}{2}\\ m\end{array}}\right) \Bigl (\dfrac{r}{n}\Bigr )^m\Biggr |\nonumber \\{} & {} \quad = \Biggl |\sum _{m=1}^{\infty }\dfrac{(-1)^m}{4^m}\dfrac{\left( {\begin{array}{c}2r+2m-2\\ r+m-1\end{array}}\right) \left( {\begin{array}{c}r+m-1\\ r-1\end{array}}\right) }{\left( {\begin{array}{c}2r-2\\ r-1\end{array}}\right) }\Bigl (\dfrac{r}{n}\Bigr )^m\Biggr |\nonumber \\{} & {} \quad \le \sum _{m=1}^{\infty } \dfrac{1}{4^m} \dfrac{\left( {\begin{array}{c}2r+2m-2\\ r+m-1\end{array}}\right) \left( {\begin{array}{c}r+m-1\\ r-1\end{array}}\right) }{\left( {\begin{array}{c}2r-2\\ r-1\end{array}}\right) }\Bigl (\dfrac{r}{n}\Bigr )^m \nonumber \\{} & {} \quad \le \sum _{m=1}^{\infty } \dfrac{2\sqrt{r-1}}{\sqrt{\pi (r+m-1)}} \left( {\begin{array}{c}r+m-1\\ r-1\end{array}}\right) \Bigl (\dfrac{r}{n}\Bigr )^m\nonumber \\{} & {} \quad \Biggl (\text {since}\ \dfrac{4^k}{2\sqrt{k}}\le \left( {\begin{array}{c}2k\\ k\end{array}}\right) \le \dfrac{4^k}{\sqrt{\pi k}}\ \forall \ k \ge 1\Biggr )\nonumber \\{} & {} \quad < \dfrac{2r}{n}\sum _{m=0}^{\infty } \left( {\begin{array}{c}r+m\\ r-1\end{array}}\right) \Bigl (\dfrac{r}{n}\Bigr )^m\nonumber \\{} & {} \quad \le \dfrac{2r}{n}\sum _{m=0}^{\infty }r^{m+1}\Bigl (\dfrac{r}{n}\Bigr )^m\ \Biggl (\text {as}\ \left( {\begin{array}{c}r+m\\ r-1\end{array}}\right) \le r^{m+1}\ \forall r \ge 1\Biggr )\nonumber \\{} & {} \quad = \dfrac{2r^2}{n}\sum _{m=0}^{\infty }\Bigl (\dfrac{r^2}{n}\Bigr )^m \le \dfrac{4r^2}{n} \ \ \text {for all}\ \ n \ge 2r^2. \end{aligned}$$

From (2.20) and (2.21), it follows that for $n \ge 2r^2$,

2.22 $$\begin{aligned}{} & {} H_r \ge \dfrac{\pi }{2}\Bigl (\dfrac{1}{2}\Bigr )_{r-1}\dfrac{1}{n^{r-\frac{1}{2}}}-\dfrac{\pi }{2}\Bigl (\dfrac{1}{2}\Bigr )_{r-1}\dfrac{4r^2}{n^{r+\frac{1}{2}}}-\dfrac{(r-1)!}{n^r}\nonumber \\{} & {} \quad > \dfrac{\pi }{2}\Bigl (\dfrac{1}{2}\Bigr )_{r-1}\dfrac{1}{n^{r-\frac{1}{2}}}-\Bigl ((r-1)!+2\pi r^2\Bigl (\dfrac{1}{2}\Bigr )_{r-1}\Bigr )\dfrac{1}{n^r}. \end{aligned}$$

This finishes the estimation of the lower bound for $H_r$.

For the upper bound estimation of $H_r$, we start with (2.19) in the following way

2.23 $$\begin{aligned}{} & {} H_r \le \dfrac{C(r)}{n^{r-\frac{1}{2}}}-\dfrac{(r-1)!}{(n+r)^r}+\sum _{k=1}^{\infty }\dfrac{1}{k\pi ^k}\Bigl (\dfrac{k}{2}\Bigr )_r \dfrac{1}{n^{r+\frac{k}{2}}}\nonumber \\{} & {} \quad < \dfrac{C(r)}{n^{r-\frac{1}{2}}}-\dfrac{(r-1)!}{(2n)^r}+\sum _{k=1}^{\infty }\dfrac{1}{k\pi ^k}\Bigl (\dfrac{k}{2}\Bigr )_r \dfrac{1}{n^{r+\frac{k}{2}}}\ \ \Bigl (\text {since},\ \dfrac{1}{(n+r)^r}> \dfrac{1}{(2n)^r}\ \forall \ n>r\Bigr )\nonumber \\{} & {} \quad = \dfrac{C(r)}{n^{r-\frac{1}{2}}}-\dfrac{(r-1)!}{(2n)^r}+\dfrac{1}{n^{r+\frac{1}{2}}}\sum _{k=0}^{2r-2}\dfrac{1}{(k+1)\pi ^{k+1}}\Bigl (\dfrac{k+1}{2}\Bigr )_r \dfrac{1}{\sqrt{n}^k}+\dfrac{1}{n^{r+\frac{1}{2}}}\sum _{k=2r}^{\infty }\dfrac{1}{k\pi ^k}\Bigl (\dfrac{k}{2}\Bigr )_r \dfrac{1}{\sqrt{n}^{k-1}}\nonumber \\{} & {} \quad \le \dfrac{C(r)}{n^{r-\frac{1}{2}}}-\dfrac{(r-1)!}{(2n)^r}+\dfrac{1}{n^{r+\frac{1}{2}}}\underset{:=\widehat{C_2}(r)}{\underbrace{\sum _{k=0}^{2r-2}\dfrac{1}{(k+1)\pi ^{k+1}}\Bigl (\dfrac{k+1}{2}\Bigr )_r \dfrac{1}{r^k}}}+\dfrac{r}{n^{r+\frac{1}{2}}}\underset{:=S(r)}{\underbrace{\sum _{k=2r}^{\infty }\dfrac{1}{k\pi ^k}\Bigl (\dfrac{k}{2}\Bigr )_r \dfrac{1}{r^k}}}\nonumber \\{} & {} \quad \Bigl (\text {since}\ \dfrac{1}{\sqrt{n}^k} \le \dfrac{1}{r^k} \forall \ n \ge r^2\Bigr ). \end{aligned}$$

In order to estimate the infinite series *S*(*r*), we need to give an upper bound of $\Bigl (\dfrac{k}{2}\Bigr )_r$ by rewriting as

$$\begin{aligned} \Bigl (\dfrac{k}{2}\Bigr )_r=\Bigl (\dfrac{k}{2}\Bigr )^r \prod _{i=0}^{r-1}\Bigl (1+\dfrac{2i}{k}\Bigr ):=\Bigl (\dfrac{k}{2}\Bigr )^r P(r,k). \end{aligned}$$

Now,

2.24 $$\begin{aligned} \log P(r,k) =\sum _{i=0}^{r-1}\log \Bigl (1+\dfrac{2i}{k}\Bigr )<\sum _{i=0}^{r-1}\dfrac{2i}{k}=\dfrac{r(r-1)}{k} \ \implies P(r,k) < e^{\frac{r(r-1)}{k}}. \end{aligned}$$

Using (2.24), we obtain

2.25 $$\begin{aligned}{} & {} S(r) < \sum _{k=2r}^{\infty }\dfrac{1}{k\pi ^k}\Bigl (\dfrac{k}{2}\Bigr )^r e^{\frac{r(r-1)}{k}} \dfrac{1}{r^k} \nonumber \\{} & {} \quad \le \dfrac{e^{\frac{r-1}{2}}}{2^r}\sum _{k=2r}^{\infty }\dfrac{k^{r-1}}{(\pi r)^k}\ \ \Bigl (\text {since}\ e^{\frac{r(r-1)}{k}} \le e^{\frac{r-1}{2}} \forall \ k \ge 2r\Bigr ). \end{aligned}$$

Moreover, $k^{r-1}< r^{k}$ for all $r \ge 2$ and $k \ge 2r$. To observe this fact, note that $k^{r-1}< r^{k}$ is equivalent to

2.26 $$\begin{aligned} \dfrac{r-1}{\log r}< \dfrac{k}{\log k}. \end{aligned}$$

Define $f(x):=\dfrac{x}{\log x}$ and observe that *f*(*x*) is strictly increasing for all $x>e$. As $k \ge 2r \ge 4 >e$, it follows that $f(k)>f(2r)$ and the fact that $f(2r) > \dfrac{r-1}{\log r}$ for $r \ge 2$, we conclude (2.26).

Applying (2.26) in (2.25), we get

2.27 $$\begin{aligned} S(r)< \dfrac{e^{\frac{r-1}{2}}}{2^r}\sum _{k=2r}^{\infty }\dfrac{1}{\pi ^k} =\dfrac{\pi }{\sqrt{e}(\pi -1)}\Bigl (\dfrac{\sqrt{e}}{2 \ \pi ^2}\Bigr )^r < \dfrac{1}{10^r}. \end{aligned}$$

Hence, by (2.27) and (2.23), we obtain for all $n \ge r^2$,

2.28 $$\begin{aligned}{} & {} H_r < \dfrac{C(r)}{n^{r-\frac{1}{2}}}-\dfrac{(r-1)!}{2^r\ n^r}+\dfrac{\widehat{C_2}(r)}{n^{r+\frac{1}{2}}}+\dfrac{r}{10^r\ n^{r+\frac{1}{2}}}\nonumber \\{} & {} \quad =\dfrac{C(r)}{n^{r-\frac{1}{2}}}-\dfrac{(r-1)!}{2^r\ n^r}+ \underset{=C_2(r)}{\underbrace{\Bigl (\widehat{C_2}(r)+\dfrac{r}{10^r}\Bigr )}}\dfrac{1}{n^{r+\frac{1}{2}}}. \end{aligned}$$

$$\square $$

## Proof of Theorem 1.6 and 1.8

### Proof of Theorem 1.6

Applying (2.13) and (2.8) in (2.7), we have for $n \ge 85=N_1(1)$,

3.1 $$\begin{aligned} L^{(1)}(n)-\dfrac{1}{n^2}<\Delta \log {\overline{p}}(n) < U^{(1)}(n)+\dfrac{1}{n^2}. \end{aligned}$$

It is straightforward to show that for $n \ge 457$,

3.2 $$\begin{aligned} -\dfrac{3}{2(n+1)}+\dfrac{\pi }{2\sqrt{n}(\widehat{\mu }(n)-1)}+ \dfrac{1}{n^2}< -\dfrac{\pi ^2}{10n} \end{aligned}$$

and therefore

3.3 $$\begin{aligned} U^{(1)}(n)+\dfrac{1}{n^2} <\dfrac{\pi }{2\sqrt{n}}-\dfrac{\pi ^2}{10n}. \end{aligned}$$

Define $c_n:=\dfrac{\pi }{2\sqrt{n}}-\dfrac{\pi ^2}{10n}$ and $d_n:=\dfrac{\pi }{2\sqrt{n}}+\dfrac{\pi ^2}{40n}$. It can be easily checked that for $n \ge 3$,

3.4 $$\begin{aligned} c_n< d_n-\dfrac{d^2_n}{2}+\dfrac{d^3_n}{3}-\dfrac{d^4_n}{4}< \log (1+d_n) \end{aligned}$$

since $\log (1+x)>x-\dfrac{x^2}{2}+\dfrac{x^3}{3}-\dfrac{x^4}{4}\ \text {for}\ x>0$. Invoking (3.3) and (3.4) in (3.1), we get for $n \ge 457$,

3.5 $$\begin{aligned} \Delta \log {\overline{p}}(n) < \log \Bigl (1+\dfrac{\pi }{2\sqrt{n}}+\dfrac{\pi ^2}{40n}\Bigr ). \end{aligned}$$

Similarly as before, it can be readily shown that for $n \ge 79$,

3.6 $$\begin{aligned} L^{(1)}(n)-\dfrac{1}{n^2}> \dfrac{\pi }{2\sqrt{n}}-\dfrac{\pi ^2}{8n}+\dfrac{\pi ^3}{24n^{3/2}} \end{aligned}$$

and

3.7 $$\begin{aligned} \dfrac{\pi }{2\sqrt{n}}-\dfrac{\pi ^2}{8n}+\dfrac{\pi ^3}{24n^{3/2}} > \log \Bigl (1+\dfrac{\pi }{2\sqrt{n}}\Bigr ) \end{aligned}$$

as $\log (1+x)<x-\dfrac{x^2}{2}+\dfrac{x^3}{3}$ for $x>0$. Applying (3.6) and (3.7) into (3.1), it follows that for $n \ge 85$,

3.8 $$\begin{aligned} \Delta \log {\overline{p}}(n) > \log \Bigl (1+\dfrac{\pi }{2\sqrt{n}}\Bigr ). \end{aligned}$$

Equations (3.5) and (3.8) conclude the proof of Theorem 1.6 except for $26 \le n \le 456$, which we confirm by numerical checking in Mathematica. $\square$

### Proof of Theorem 1.8

Applying (2.8) and (2.16) to the lower bound of (2.7), it follows that for $n \ge \max \{N_1(r), 2r^2\}$,

3.9 $$\begin{aligned} (-1)^{r-1}\Delta ^r \log {\overline{p}}(n)>\dfrac{C(r)}{n^{r-\frac{1}{2}}}-\dfrac{C_1(r)}{n^r}-\dfrac{1}{n^{r+1}}>\dfrac{C(r)}{n^{r-\frac{1}{2}}}-\dfrac{1+C_1(r)}{n^r}. \end{aligned}$$

We recall from (1.10) that $N_2(r)=\Biggl \lceil \Biggl (\dfrac{1+C_1(r)}{C(r)}\Biggr )^2\Biggr \rceil$. Then for all $n \ge \max \{N_1(r),2r^2,N_2(r)\}$, it follows that

3.10 $$\begin{aligned} (-1)^{r-1}\Delta ^r \log {\overline{p}}(n)> \dfrac{C(r)}{n^{r-\frac{1}{2}}}-\dfrac{1+C_1(r)}{n^r}> \log \Biggl (1+\dfrac{C(r)}{n^{r-\frac{1}{2}}}-\dfrac{1+C_1(r)}{n^r}\Biggr )>0. \end{aligned}$$

For the upper bound estimation, putting (2.8) and (2.16) together into the upper bound of (2.7), it follows that for $n \ge \max \{N_1(r), 2r^2\}$,

3.11 $$\begin{aligned} (-1)^{r-1}\Delta ^r \log {\overline{p}}(n)< & {} \dfrac{C(r)}{n^{r-\frac{1}{2}}}-\dfrac{(r-1)!}{2^r\ n^r}+\dfrac{C_2(r)}{n^{r+\frac{1}{2}}}+\dfrac{1}{n^{r+1}}\nonumber \\< & {} \dfrac{C(r)}{n^{r-\frac{1}{2}}}-\dfrac{(r-1)!}{2^r\ n^r}+\dfrac{C_2(r)+1}{n^{r+\frac{1}{2}}}. \end{aligned}$$

Next, our goal is to show for $n \ge N_3(r)$,

$$\begin{aligned} -\dfrac{(r-1)!}{2^r\ n^r}+\dfrac{C_2(r)+1}{n^{r+\frac{1}{2}}} < -\dfrac{C(r)^2}{2\ n^{2r-1}}, \end{aligned}$$

which is equivalent to

3.12 $$\begin{aligned} \dfrac{C(r)^2}{2}< n^{r-1}\Biggl [\dfrac{(r-1)!}{2^r}-\dfrac{C_2(r)+1}{\sqrt{n}}\Biggr ]. \end{aligned}$$

Note that for all $n \ge \Biggl \lceil \Biggl (\dfrac{2^{r+1}\Bigl (C_2(r)+1\Bigr )}{(r-1)!}\Biggr )^2\Biggr \rceil$, $\dfrac{(r-1)!}{2^{r+1}}-\dfrac{C_2(r)+1}{\sqrt{n}}>0$ and therefore

3.13 $$\begin{aligned}{} & {} n^{r-1}\Biggl [\dfrac{(r-1)!}{2^r}-\dfrac{C_2(r)+1}{\sqrt{n}}\Biggr ]= n^{r-1}\Biggl [\dfrac{(r-1)!}{2^{r+1}}\nonumber \\{} & {} \quad +\dfrac{(r-1)!}{2^{r+1}}-\dfrac{C_2(r)+1}{\sqrt{n}}\Biggr ]>n^{r-1} \dfrac{(r-1)!}{2^{r+1}}. \end{aligned}$$

Hence, to prove (3.12), it is sufficient to prove

3.14 $$\begin{aligned} n^{r-1} \dfrac{(r-1)!}{2^{r+1}} > \dfrac{C(r)^2}{2} \ \text {which holds for all}\ \ n \ge \Biggl \lceil \root r-1 \of {\Biggl (\dfrac{2^r C(r)^2}{(r-1)!}\Biggr )} \Biggr \rceil . \end{aligned}$$

Recall that

$$\begin{aligned} N_3(r)=\max \Biggl \{N_1(r),2r^2, \Biggl \lceil \Biggl (\dfrac{2^{r+1}\Bigl (C_2(r)+1\Bigr )}{(r-1)!}\Biggr )^2\Biggr \rceil , \Biggl \lceil \root r-1 \of {\Biggl (\dfrac{2^r C(r)^2}{(r-1)!}\Biggr )} \Biggr \rceil \Biggr \}\ \ (\text {cf.}\ (1.11). \end{aligned}$$

From (3.11) and (3.12), it follows that for $n \ge N_3(r)$,

3.15 $$\begin{aligned} (-1)^{r-1}\Delta ^r \log {\overline{p}}(n)< \dfrac{C(r)}{n^{r-\frac{1}{2}}}-\dfrac{C(r)^2}{2\ n^{2r-1}}< \log \Bigl (1+\dfrac{C(r)}{n^{r-1/2}}\Bigr ). \end{aligned}$$

Equations (3.10) and (3.15) together imply that for $n \ge \max \{N_2(r),N_3(r)\}=N(r)$, (1.13) holds. $\square$

## Data Availability

We hereby confirm that Data sharing not applicable to this article as no datasets were generated or analyzed during the current study.
